# A novel compound which sensitizes BRAF wild-type melanoma cells to vemurafenib in a TRIM16-dependent manner

**DOI:** 10.18632/oncotarget.10700

**Published:** 2016-07-19

**Authors:** Selina K. Sutton, Daniel R. Carter, Patrick Kim, Owen Tan, Greg M. Arndt, Xu Dong Zhang, Jonathan Baell, Benjamin D. Noll, Shudong Wang, Naresh Kumar, Grant A. McArthur, Belamy B. Cheung, Glenn M. Marshall

**Affiliations:** ^1^ Children's Cancer Institute for Medical Research, Lowy Cancer Research Centre, University of New South Wales, New South Wales, Australia; ^2^ School of Women's and Children's Health, UNSW Australia, New South Wales, Australia; ^3^ Priority Research Centre for Cancer Research Oncology and Immunology Unit, University of Newcastle, New South Wales, Australia; ^4^ Department of Medicinal Chemistry, Faculty of Pharmacy and Pharmaceutical Sciences, Monash Institute of Pharmaceutical Sciences, Monash University, Victoria, Australia; ^5^ Centre for Drug Discovery and Development, Sansom Institute for Health Research and School of Pharmacy and Medical Sciences, University of South Australia, South Australia, Australia; ^6^ School of Chemistry, University of New South Wales Australia, New South Wales, Australia; ^7^ Translational Research Laboratory, Peter MacCallum Cancer Centre, Victoria, Australia; ^8^ Kids Cancer Centre, Sydney Children's Hospital, New South Wales, Australia

**Keywords:** BRAF inhibitor, melanoma, TRIM16, vemurafenib, novel compound

## Abstract

There is an urgent need for better therapeutic options for advanced melanoma patients, particularly those without the BRAF^V600E/K^ mutation. In melanoma cells, loss of TRIM16 expression is a marker of cell migration and metastasis, while the BRAF inhibitor, vemurafenib, induces melanoma cell growth arrest in a TRIM16-dependent manner. Here we identify a novel small molecule compound which sensitized BRAF wild-type melanoma cells to vemurafenib. High throughput, cell-based, chemical library screening identified a compound (C012) which significantly reduced melanoma cell viability, with limited toxicity for normal human fibroblasts. When combined with the BRAF^V600E/K^ inhibitor, vemurafenib, C012 synergistically increased vemurafenib potency in 5 BRAF^WT^ and 4 out of 5 BRAF^V600E^ human melanoma cell lines (Combination Index: CI < 1), and, dramatically reduced colony forming ability. In addition, this drug combination was significantly anti-tumorigenic *in vivo* in a melanoma xenograft mouse model. The combination of vemurafenib and C012 markedly increased expression of TRIM16 protein, and knockdown of TRIM16 significantly reduced the growth inhibitory effects of the vemurafenib and C012 combination. These findings suggest that the combination of C012 and vemurafenib may have therapeutic potential for the treatment of melanoma, and, that reactivation of TRIM16 may be an effective strategy for patients with this disease.

## INTRODUCTION

Melanoma is a highly aggressive malignancy, and although it accounts for only a small percentage of skin cancers overall, it is responsible for the majority of skin cancer deaths [1]. Furthermore, melanoma is an increasing clinical problem with the global incidence of the disease rising, particularly amongst young [2] and middle-aged adults [3]. While early detection and removal by surgical excision results in high cure rates, patients with metastatic melanoma have limited effective treatment options available. Approximately 50% of melanoma patients harbour activating mutations in the v-raf murine sarcoma viral oncogene homolog B1 (BRAF) protein, most commonly BRAF^V600E^, resulting in MAPK pathway activation, while another 15–20% of patients possess mutations in neuroblastoma RAS viral (v-ras) oncogene homolog (NRAS), activating both the MAPK and PI3K pathways [1, 4]. Patients with the NRAS mutation (mutually exclusive to BRAF mutations) have a worse prognosis than BRAF mutant patients [5]. The remaining patients that have neither gene mutation typically have de-regulated kinases including amplification and overexpression of PAK1 [6, 7], inactivation of the NF-1 tumor suppressor [8], loss of NF-1 associated with RAS activation [9, 10] and cell cycle aberrations such as CCND1 and CDK4 amplification [11].

As melanomas are deregulated via multiple pathways and are able to by-pass mutant BRAF inhibitors to reactivate oncogenic signalling [12–14], combination treatment is essential to effectively treat the disease. Therefore, an increased knowledge of the molecular pathology of melanoma is required to understand how drug combinations might contribute to an increased anti-cancer signal. Determination of patient subgroups that are likely to respond to treatment is also important [15–17]. Whilst the advent of targeted therapies in the form of BRAF inhibitors has yielded clinically meaningful benefit for patients harbouring the BRAF mutation, patients that do not have a druggable mutant kinase have few targeted therapy options available. To date, there are no Food and Drug Administration (FDA) approved targeted therapies for NRAS mutant melanoma patients [18]. In clinical trials, only one targeted therapy in the form of a MEK inhibitor, binimetinib (MEK162), is currently being investigated for NRAS^Q61^ mutant patient treatment vs dacarbazine (Clinical trial NCT01763164). The limited availability of therapeutic options highlight BRAF^WT^/NRAS mutant patients as a subgroup most in need of new therapeutic treatment strategy and a better understanding of disease pathology.

Treatment modalities for metastatic melanoma involve immunotherapy, such as nivolumab and ipilumamib [19]. However, identification of patient sub-groups that are likely to respond to immune based therapy is still largely unknown [20, 21]. This emphasises the important role targeted therapies have to play in melanoma treatment. Targeted BRAF inhibitor, vemurafenib, was approved for BRAF^V600mut^ patients by the FDA in 2011 and is in frequent clinical use. Vemurafenib has been shown to improve median progression free survival (6.9 vs 1.6 months) and median overall survival (13.6 vs 9.7 months) in patients compared to the previous standard of care, dacarbazine [21]. Recent data has clearly indicated the benefit of combining BRAF inhibitors with MEK-inhibitors with the combination of dabrafenib, trametinib, vemurafenib and cobimetinib improving overall survival compared to BRAF-inhibitor monotherapy [22–24]. Treatment of NRAS^Q61^ melanoma with the MEK inhibitor, binimetinib (MEK162), is currently in clinical trial and is the only targeted treatment for NRAS mutant melanomas. There is an additional need for novel therapies and approaches to the treatment of BRAF wild-type melanomas including NRAS mutant melanoma. Moreover even in BRAF mutant melanoma the vast majority of patients still develop progressive disease after combined BRAF and MEK-inhibition [21]. Thus, improvements in the effectiveness of these targeted therapies in the form of synergistic combination treatments are urgently required.

Identification of tumor suppressor proteins in melanoma is informative of the steps in melanomagenesis as it progresses from normal nevus to a malignant melanoma. Members of the tripartite motif (TRIM) family of proteins have been implicated in the pathogenesis of numerous cancers, both as oncoproteins and tumor suppressor proteins [25]. These proteins typically carry a characteristic RING b-box-Coiled-coil organisational structure [25, 26]. We have previously reported that the expression of TRIM16 is significantly reduced *in vivo* during the transition of normal skin to squamous cell carcinoma (SCC), while increased expression of this protein reduces SCC cell migration *in vitro* [27]. In addition, we have observed that TRIM16 can act as a tumor suppressor in the childhood cancer neuroblastoma [28], via the binding and down-regulation of cytoplasmic vimentin and nuclear E2F1 [28]. In melanoma, we have shown TRIM16 protein expression to be significantly associated with favourable prognosis in stage III melanoma patients, while enforced expression identified TRIM16 as a growth and metastasis suppressor, its effect mediated via IFNβ1 transcriptional activation [29]. Moreover, we showed high levels of TRIM16 in melanoma tissues from patients treated with vemurafenib correlated with clinical response. These data suggest the potential of TRIM16 as a candidate tumor suppressor protein in melanoma and that restoration of TRIM16 expression may be a potential therapeutic strategy for melanoma treatment.

In this study, we have identified an anti-melanoma compound (C012) through high throughput chemical library screening. We have shown that C012 synergises with vemurafenib, targeting both BRAF wild-type and mutant cells. The combination of C012 with vemurafenib decreased melanoma cell viability, and increased TRIM16 protein expression *in vitro* and *in vivo*. TRIM16 expression was required for the maximal C012/vemurafenib combination efficacy *in vitro*. Overall, our findings have identified a novel small molecule with clinical potential in the treatment of melanoma, including BRAF^WT^ melanoma, and provide mechanistic insights into the role of TRIM16 in this process.

## RESULTS

### Identification of C012 as an anti-melanoma compound

**Figure 1:**
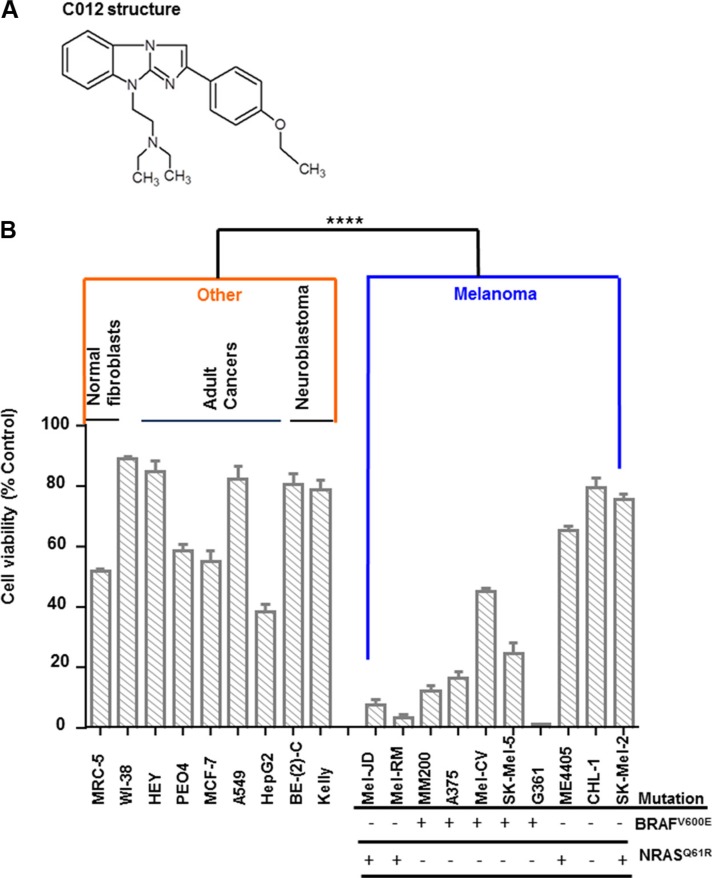
Compound 012 (C012) has single agent activity in melanoma cell lines (**A**) 2-(2-(4-ethoxyphenyl)-9H-benzo [d]imidazo[1, 2-a]imidazol-9-yl)-N, N diethylethanamine dihydrochloride (Compound 012 (C012)) chemical structure is shown. (**B**) A panel of normal fibroblasts (WI-38, MRC-5), adult cancer (HEY, PE04, MCF-7, A549, HEPG2), neuroblastoma (BE-(2)-C, Kelly), and melanoma (Mel-JD, Mel-RM, MM200, A375, SK-Mel-5, G361, ME4405, CHL-1 and SK-Mel-2) cell lines were treated with 10 μM of C012 for 72 hours and cell viability was measured using the Alamar Blue assay. Cell viability is expressed as percentage of untreated control cells. The BRAF and NRAS mutational status of the panel of melanoma cells lines is also indicated. Average viability of melanoma cell lines (blue) vs the other cancer and normal fibroblasts (orange) were analysed by the Student's *t*-test, mean + SEM. ****P < 0.0001.

A high throughput screening of a 10,560 compound library subset from the Walter and Eliza Hall Institute, for enhancers of the HDAC inhibitor, SAHA, identified three candidate compounds that had single agent activity towards a panel of melanoma cell lines. The compound that was most efficacious over a range of melanoma cells was identified as 2-(2-(4-ethoxyphenyl)-9*H*-benzo [d]imidazo[1, 2-a]imidazol-9-yl)-*N*, *N*-diethylethanamine (Figure 1A), subsequently referred to as C012. To determine whether C012 had specific cytotoxicity to melanoma cells over other cancer cells, C012 was screened at 10 μM as a single agent for its effects on cell viability using the Alamar Blue assay against a panel of human cancer cell lines (neuroblastoma, breast, ovarian, lung, liver), normal human fibroblasts (MRC-5 and WI-38) and 10 human melanoma cell lines (Figure 1B). C012 demonstrated marked single agent activity against melanoma cells, but showed reduced toxicity towards normal fibroblasts and other cancer cell lines (*P* = 0.0001, Figure 1B). This toxicity was not dependent on the BRAF and NRAS mutational status, as indicated in Figure 1B. Thus, the cytopathic effect of C012 as a single agent was greater for melanoma cells compared to other cancer types or normal fibroblasts.

### C012 synergises with vemurafenib in BRAF wild-type and mutant cell lines

**Figure 2:**
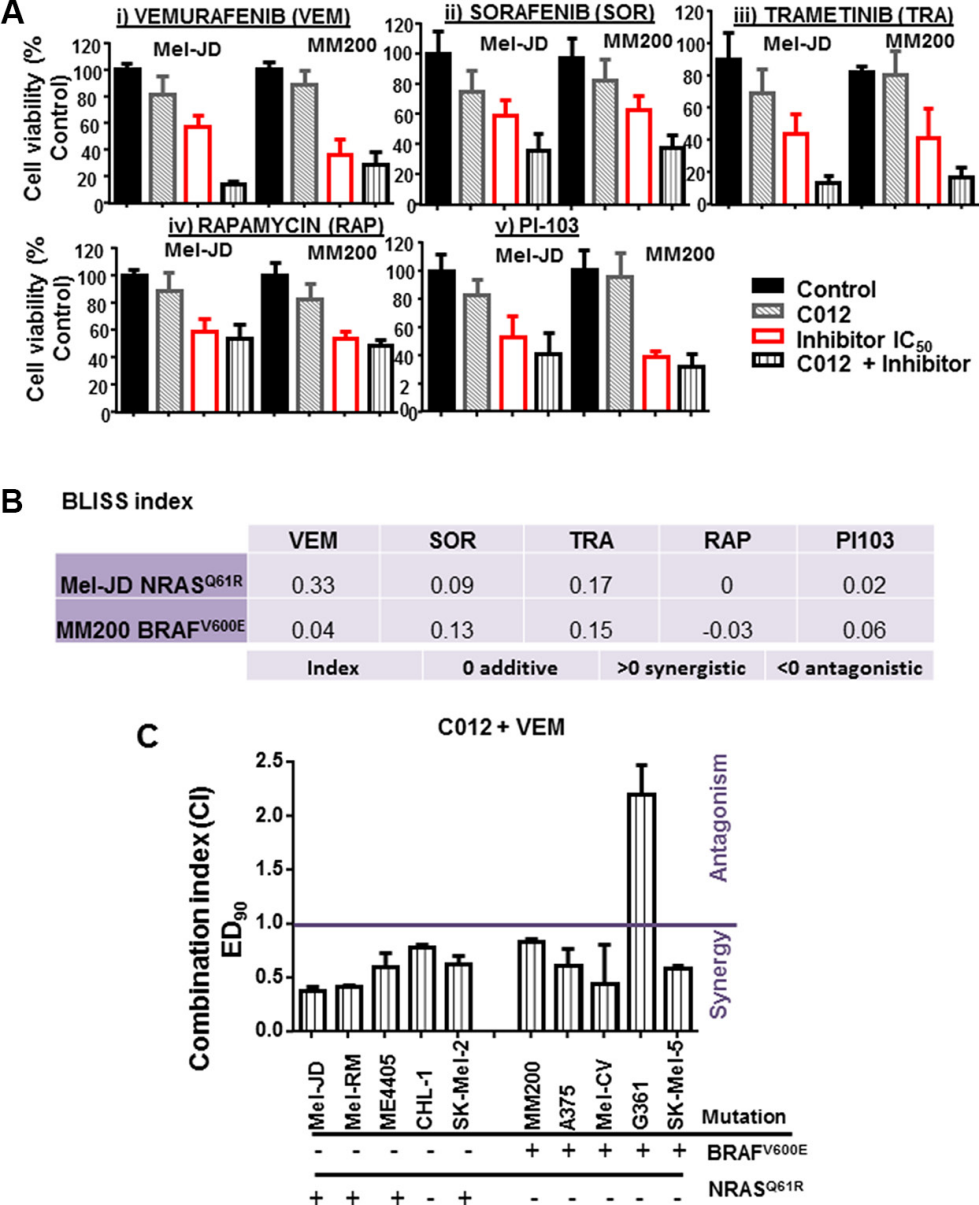
Vemurafenib is synergistic with C012 in melanoma cells (**A**) Mel-JD (BRAF^WT^/NRAS^Q61R^) or MM200 (NRAS^WT^/BRAF^V600E^) cells were treated with C012 at 4 μM and either vemurafenib (VEM) (i), sorafenib (SOR) (ii), trametinib (TRA) (iii), rapamycin (RAP) (iv) or PI-103 (v) at IC_50_ doses to determine cell viability of the compounds with C012 in combination. (**B**) The Bliss additivity index was applied to determine combination effect. The (additive = 0, synergistic > 0 or antagonistic < 0) relationship between the respective inhibitor and C012 is shown. (**C**) A panel of melanoma cell lines was assessed for drug synergy at optimised doses based on the IC_50_ dose of C012 and vemurafenib of each line. Synergy analysis was determined by the Calcusyn algorithm at ED_90_. Statistical analysis was performed by the Student's *t*-test. **P < 0.01.

Currently, single agent treatment of metastatic melanoma leads to only transient tumor response; therefore, C012 was tested for potential synergistic activity in combination with clinically approved agents in the treatment of melanoma. These include the MAPK pathway targeting agents: vemurafenib, sorafenib and trametinib. We also combined C012 with the PI3K inhibitor, PI-103, and rapamycin, targeting the mTOR signal, which has been shown to have pre-clinical significance in preventing melanoma tumor growth [30]. C012 was used at an IC_20_ dose of 4 μM, in combination with these drugs at IC_50_ concentrations against Mel-JD and MM200 cell lines (Figure 2A). Cell viability was assessed using the Alamar Blue assay (Figure 2A). These cell lines were selected as they exhibited similar cytotoxicity to C012 treatment (Figure 1B) and represented the commonest mutant melanoma pathologies: BRAF^WT^/NRAS^Q61R^ (Mel-JD) and BRAF^V600E^/NRAS^WT^ (MM200). Notably, agents that targeted the MAPK pathway (vemurafenib, sorafenib and trametinib) were the most effective in combination with C012 in both cell lines (Figure 2A i–iii) determined by their respective Bliss additivity score (Figure 2B). Combination of C012 with PI-103 or rapamycin yielded a marginally significant reduction in cell viability in Mel-JD and MM200 cell lines (Figure 2A iv–v) and low additive BLISS scores (Figure 2B). Combination C012 and vemurafenib treatment of Mel-JD cells yielded a 4-fold decrease in cell viability (Figure 2A i) and showed the highest BLISS synergy index of 0.33 (Figure 2B).

To further assess the synergistic relationship between C012 and vemurafenib, drug combination synergy studies were undertaken using the IC_50_ dose of vemurafenib and C012 for each melanoma line and performing a constant ratio dose range with assessment of cell viability (Figure 2C). Synergy was determined by using the Calcusyn algorithm and represented by the combination index (CI) values at ED_90_ where a CI < 0.9 indicates a synergistic relationship and CI values > 1 indicate drug antagonism [31]. Synergy at greater cytopathic drug concentrations (ED_90_ and above) is considered more therapeutically relevant than ED_50_ synergy values [31]. At ED_90_ concentrations, synergy was observed in all 5 BRAF^WT^ melanoma cell lines and in 4/5 BRAF^V600E^ melanoma lines (Figure 2C). These results demonstrate efficacy of combination C012 and vemurafenib against a range of melanomas, irrespective of BRAF/NRAS mutational status.

In order to assess the effect of combination treatment on normal cells, standardised doses of C012 and vemurafenib were used to treat normal fibroblasts, WI-38 and MRC-5 and normal human epidermal melanocytes (NHEM). Combination treatment resulted in only minor fold changes in cell viability compared to control and single agent treatment (Supplementary Figure 1A i–iii). BLISS additivity scores revealed no synergy with combination treatment in non-malignant cells (Supplementary Figure 1A iv).

**Figure 3:**
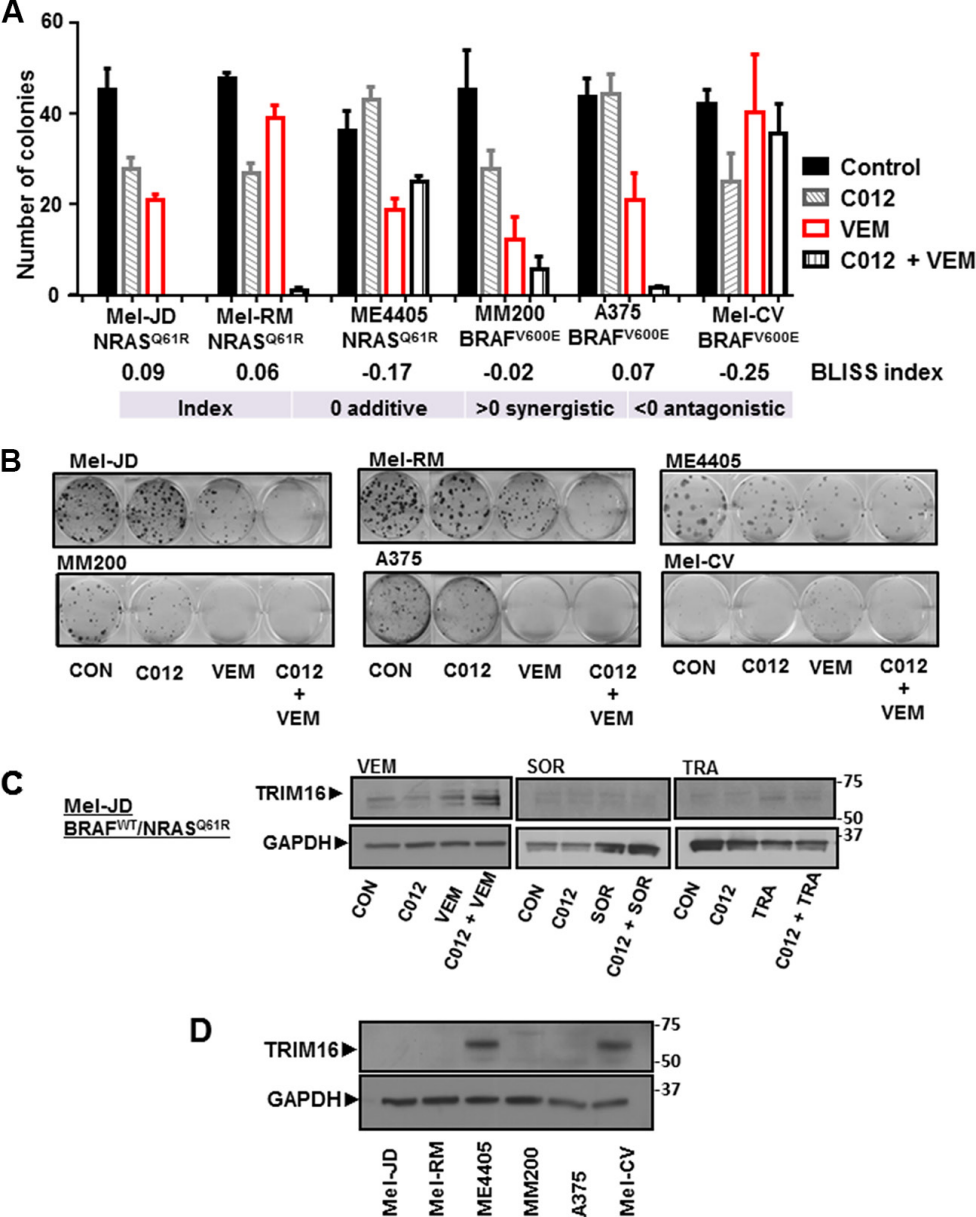
Combination of C012 and vemurafenib induces TRIM16 protein expression (**A**) The combination effect of C012 and VEM was assessed by clonogenicity assays in Mel-JD, Mel-RM, and ME4405 (BRAF^WT^/NRAS^Q61R^) and MM200, A375, and Mel-CV (NRAS^WT^/BRAF^V600E^) cell lines with standardised doses of C012 and VEM. Drug treatment was for 14 days. Bliss additivity analysis was used to determine synergy where additive relationship = 0, synergistic > 0 or antagonistic < 0 (**B**) Colonies are stained with crystal violet and counted. A cell colony is defined as > 50 cells. (**C**) Inhibitors, VEM, SOR, TRA, were assessed in combination with C012 for TRIM16 reactivation in Mel-JD cells by immunoblotting after 72 hours treatment. (**D**) Endogenous whole cell lysate from a panel of melanoma cell lines was immunoblotted for endogenous TRIM16 protein expression. GAPDH serves as a loading control.

To further assess the potential for synergy between C012 and vemurafenib, clonogenicity assays were performed. For this study, three BRAF^WT^/NRAS^Q61R^ melanoma cell lines (Mel-JD, Mel-RM and ME4405) and three BRAF^V600E^/NRAS^WT^ melanoma cell lines (MM200, A375 and Mel-CV) were treated with C012/vemurafenib as single agents or in combination. Four of six cell lines exhibited a significant reduction in colony forming ability as indicated by the Bliss additivity scores (Figure 3A and 3B). Two cell lines, ME4405 and Mel-CV were resistant to the C012/vemurafenib combination treatment and displayed antagonism as assessed by a Bliss additivity score of −0.17 (ME4405) and −0.25 (Mel-CV) (Figure 3A). Thus, sensitivity or resistance to the combination did not associate with mutant BRAF or NRAS status in melanoma cell lines.

### The combination of C012 and vemurafenib reactivates TRIM16 expression in BRAF^WT^/NRAS^Q61R^ melanoma cells

Deregulation of the MAPK and PI3K pathways are central to melanoma pathogenesis [30, 32]. Both BRAF and NRAS mutations are known to deregulate the MAPK pathway, functioning as mutually exclusive aberrations in melanoma not exposed to BRAF-inhibitors. NRAS mutant melanoma cells treated with vemurafenib exhibit a paradoxical activation of the MAPK pathway due to CRAF/BRAF dimerisation and CRAF transactivation [33, 34]. To understand the mechanism of action of the C012 and vemurafenib combination, we used immunoblotting of proteins in two BRAF^WT^/NRAS^Q61R^ (Mel-JD and Mel-RM) and two NRAS^WT^/BRAF^V600E^ (MM200 and A375) melanoma cell lines treated with combination C012/vemurafenib to determine if the drug combination altered MAPK pathway activity. We found no significant change in levels of MEK and ERK phosphorylation with the C012/vemurafenib combination treatment compared to single agent vemurafenib treatment of BRAF^WT^/NRAS^Q61R^ (Mel-JD and Mel-RM) cells (Supplementary Figure 2A) which instead displayed the expected MAPK pathway activation with vemurafenib treatment. In contrast, NRAS^WT^/BRAF^V600E^ (MM200 and A375) cells showed the expected decrease in MEK and ERK phosphorylation (Supplementary Figure 2). These data suggest that the anti-melanoma effect of combination C012 and vemurafenib was not due to altered signalling of the MAPK pathway.

TRIM16 has been recently identified as a candidate tumor suppressor gene in melanoma and is reactivated by vemurafenib in BRAF^V600E^ melanoma cells [29]. We performed immunoblotting to assess TRIM16 protein expression following C012, vemurafenib or C012 and vemurafenib combination treatment of melanoma cell lines. We showed an increase in TRIM16 protein expression in the two BRAF^WT^/NRAS^Q61R^ cell lines, Mel-JD cells (Figure 3C) and Mel-RM, with the combination treatment (Supplementary Figure 2A). TRIM16 protein levels increased with vemurafenib alone treatment of MM200 and A375 cell lines, but not with combination treatment (Supplementary Figure 2A). To determine if the increase in TRIM16 protein was specific to the combination of C012 and vemurafenib, we assessed inhibitors, sorafenib, and trametinib for TRIM16 reactivation when combined with C012 (Figure 3C). We found that the combination of C012 and vemurafenib, but not C012 and trametinib or C012 and sorafenib, was effective at reactivating TRIM16 (Figure 3C). Interestingly, on examination of endogenous expression of TRIM16 protein, we observed that the two cell lines that were most resistant to the combination treatment, ME4405 and Mel-CV (Figure 2A and 2B) also had the highest endogenous expression levels of TRIM16 (Figure 3D). TRIM16 protein did not increase in the control normal fibroblasts, MRC-5, and there was no observed change in ERK phosphorylation in the fibroblasts (Supplementary Figure 2B).

### The combination of C012 and vemurafenib requires TRIM16 protein expression

**Figure 4:**
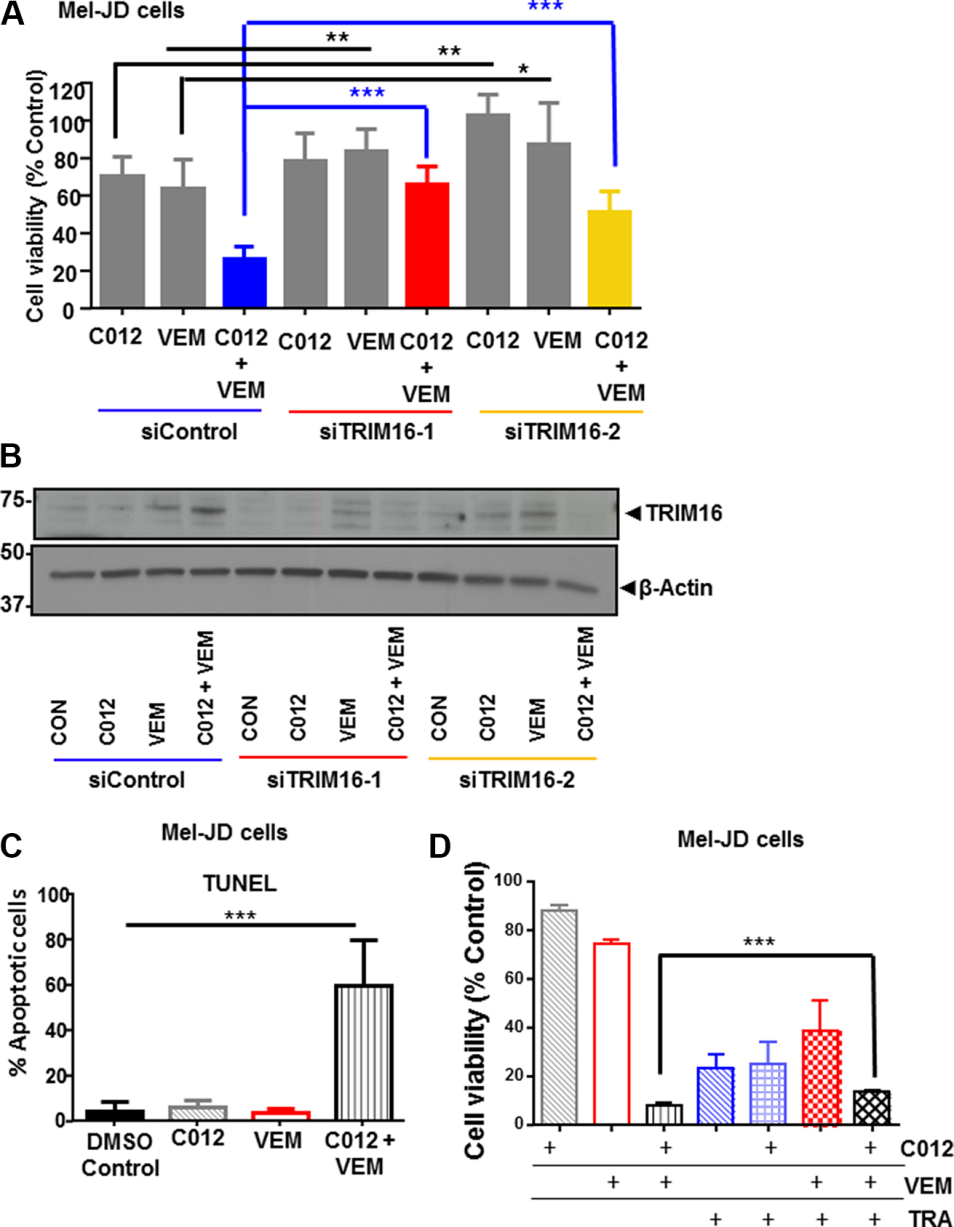
TRIM16 is partially required for combination of C012 and vemurafenib reduction in cell viability (**A**) Mel-JD cells were transfected with control siRNA or two specific TRIM16 siRNAs (TRIM16-1 and TRIM16-2) for 24 hours before DMSO control, C012 (4 μM), VEM (5 μM) or combination treatment for an additional 48 hours. Cell viability was assessed by Alamar Blue and expressed as percentage of DMSO control cells. (**B**) Western blotting was used to determine the corresponding protein expression of TRIM16 with GAPDH as a loading control. (**C**) Mel-JD cells were treated as indicated for 72 hours and apoptosis measured using the TUNEL assay. (**D**) Mel-JD cells were treated with standardized doses of C012 and VEM and an IC_80_ dose of TRA as indicated and cell viability was measured by the Alamar Blue assay. Data represents mean + SEM and was analysed by the Student's *t*-test ***P < 0.001, **P < 0.01, *P < 0.05.

We have previously shown that TRIM16 is significantly decreased during melanoma tumorigenesis and is a candidate tumor suppressor in metastatic melanoma [29]. To determine whether increased TRIM16 expression mediated the cytopathic effects of combination C012 and vemurafenib treatment, we used two TRIM16-specific siRNAs to knockdown TRIM16 for 24 hours and then treated the two BRAF^WT^/NRAS^Q61R^ (Mel-JD and Mel-RM) cell lines with the C012/vemurafenib combination for an additional 48 hours. We found that siRNAs specific to TRIM16 inhibited the cytopathic effects of the drug combination in Mel-JD (***P < 0.001) (Blue lines, Figure 4A) and Mel-RM (***P < 0.001) (Blue lines, Supplementary Figure 3A) cell lines. Single agent C012 and VEM also reached significance (**P < 0.01), but to a lesser degree than combination treatment (Figure 4A). Immunoblotting confirmed that the induction of TRIM16 protein expression by combination treatment of Mel-JD (Figure 4B) and Mel-RM (Supplementary Figure 3B) cell lines was lost with TRIM16 siRNA transfection. These data indicated that TRIM16 induction by combination treatment is partially required for the cytopathic combination drug effect. As TRIM16 protein expression is known to induce apoptosis in neuroblastoma [35], we overexpressed TRIM16 in low TRIM16 expressing melanoma cell lines, Mel-JD, A753 and G361 and showed an increase in apoptosis in the TRIM16 overexpressing cells compared to empty vector controls (*P* < 0.05) (Supplementary Figure 4A). We next showed that the combination treatment induced apoptosis in Mel-JD and Mel-RM cells as assessed by TUNEL assay in Mel-JD cells (***P < 0.001) (Figure 4C) and Mel-RM cells (*P < 0.05) (Supplementary Figure 3C). To determine whether activation of the MAPK pathway was required for cytopathic effects of the combination, the MEK inhibitor, trametinib, was used to block the MAPK pathway and cell viability was assessed in Mel-JD cells (Figure 4D). Addition of trametinib had a modest but significant inhibitory effect on the cytopathic activity of C012 and vemurafenib combination therapy (*P* < 0.001).

Since TRIM16 is a regulator of IFNβ1 transcription [29], we used IFNβ1-specific siRNA to determine whether IFNβ1 transcription was necessary for the cytopathic effects of the combination treatment. We showed that IFNβ1 is also partially required for the cytopathic effect of the combination. (***P < 0.001) (Supplementary Figure 4B). Incomplete loss of TRIM16 protein (Figure 4B) despite siRNA against TRIM16 may be explained by the ability of VEM to stabilise TRIM16 protein [29]. Collectively, these data suggest that TRIM16 and IFNβ1 activation are important to the drug combination mechanism of action.

### Combination of C012 and vemurafenib treatment is anti-tumorigenic

To determine the potential application of C012 use *in vivo*, we performed microsomal stability assays for assessment of oral administration, serum half-life studies for compound administration intravenously, and determination of its maximum tolerated dose. We found the microsomal stability of C012 to be low at 13.5 minutes (Supplementary Figure 5A). We also assessed the pharmacological properties of C012 by intravenous administration and found the plasma half-life to be a favourable 70 minutes with a C_max_ of 1761 ng/mL (*N* = 3) (Supplementary Figure 5B) and indicated this to be a more effective route of C012 administration. We determined the maximum tolerated dose of C012 to be 15 mg/kg (Supplementary Table 1A and 1B).

**Figure 5:**
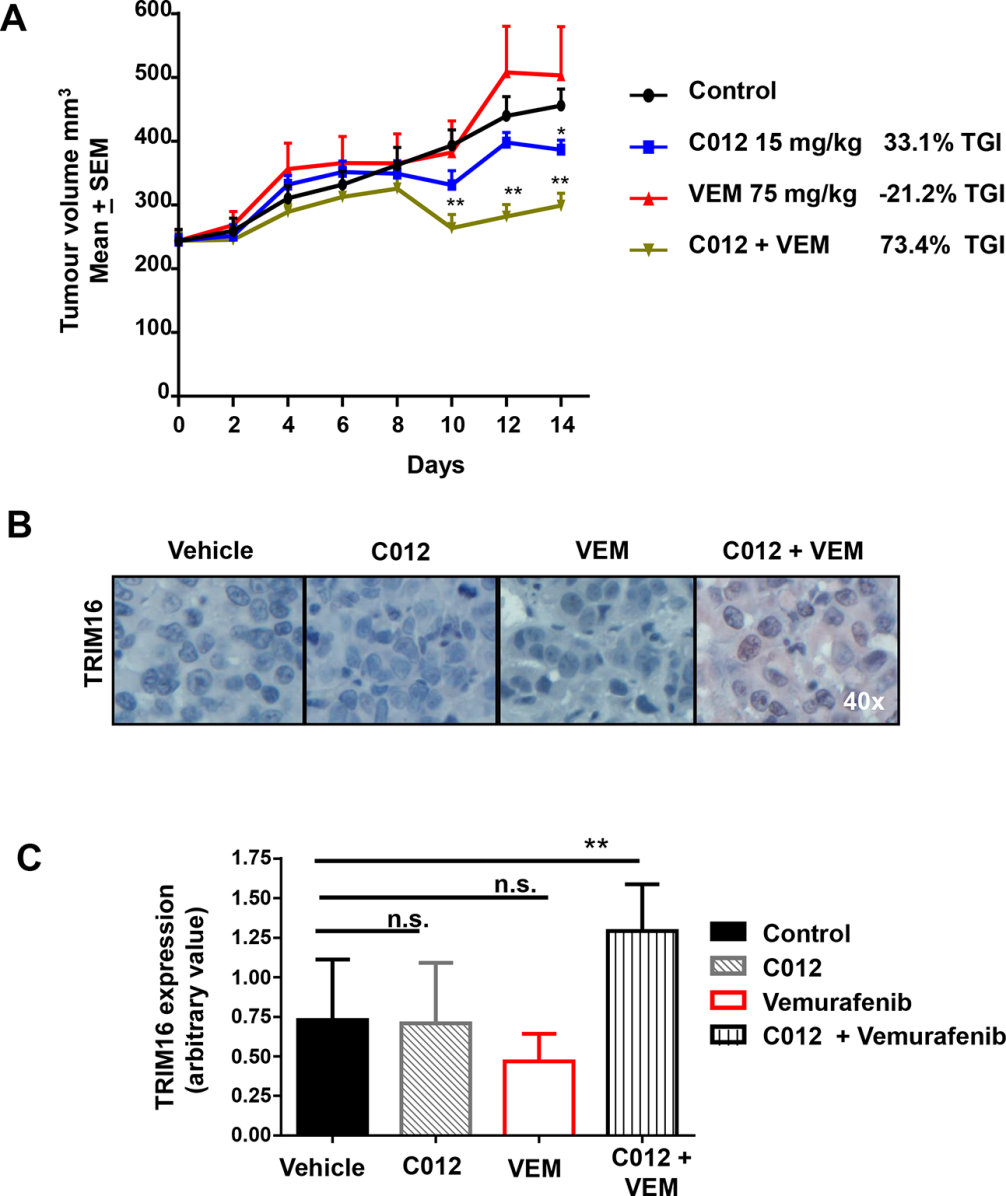
Combination of C012 and vemurafenib shows a decrease in tumor volume and increase in TRIM16 *in vivo* (**A**) Female, 5 week old Balb-(c) nude mice were engrafted with 2 × 10^6^ Mel-JD melanoma cells. A tumor volume of 250 mm^3^ was established before randomization into DMSO control, C012 at 15 mg/kg (intravenous), vemurafenib (VEM) at 75 mg/kg (oral gavage) or combination treatment groups (*N* = 8/group). Dosing was administered 5 days on, 2 days off for 21 days. Day 0-14 is shown and the tumor growth inhibition (TGI) calculated. Tumors were excised at 21 days. (**B**) Immunohistochemistry for TRIM16 expression was performed on excised tumors. TRIM16 expression can be seen with positive red chromogen staining. Representative examples are shown. (**C**) TRIM16 expression was quantified by assigning a staining intensity value by assessment of the average of three sections from each tumor sample. Each section contained at approximately 100 cells. The histogram bars represent 8 individual tumor samples assessed from each treatment group. Data represents mean + SEM and was analysed by the Student's *t*-test **P < 0.01, *P < 0.05.

*In vivo* anti-tumor efficacy of C012 as a single agent and combining with vemurafenib was evaluated against a Mel-JD xenograft model. While C012 at a dose of 15 mg/kg showed a modest anti-tumor activity with a tumor growth inhibition (TGI) of 33%, its combination treatment significantly decreased tumor growth between days 8–10, resulting in an overall TGI of 73.4% compared to control **P < 0.01 (Figure 5A). Immunohistochemical analysis of tumor samples showed a significant increase in TRIM16 protein expression in the combination treated group after 21 days of treatment with representative immunohistochemistry for red chromogen TRIM16 staining is shown for each group (Figure 5B). Semi-quantitative histological scoring data from all mice representing 8 mice per treatment group (Figure 5C) showed a statistically significant increase (**P < 0.01) confirming the enhanced level of TRIM16 expression. These data demonstrate that combination therapy with C012 and vemurafenib had *in vivo* efficacy against melanoma cells and suggest that induction of TRIM16 may be a necessary component of the therapeutic effect in the Mel-JD BRAF^WT^/NRAS^Q61R^ melanoma subtype.

## DISCUSSION

In this study, we have identified a small molecule, C012, which can synergistically promote the potency of vemurafenib to increase drug efficacy in BRAF^WT^ and BRAF^V600E^ melanoma cells. This synergistic relationship was displayed by reduction in cell viability, colony forming ability and by the induction of apoptosis in cells treated with the drug combination. Mechanistically, this combination effect is mediated, in part, by the re-activation of TRIM16 expression, previously shown to be a metastasis suppressor in melanomagenesis that can be restored by vemurafenib in BRAF^V600^ melanoma *in vitro* and in patients treated with vemurafenib [29].

We found that C012 combined most strongly with vemurafenib and other MAPK pathway inhibitors (sorafenib and trametinib), but with marginal combination effect with AKT and mTOR pathway inhibitors (PI-103 and rapamycin) suggesting C012 synergistically promotes cytotoxicity with MAPK pathway targeting agents. Human melanocyte and fibroblast lines did not undergo any appreciable increased cell death with combination treatment suggesting a likely therapeutic index. More intensive investigation is required to understand the full molecular profile of C012 and vemurafenib particularly which melanoma subtype that this combination may benefit.

Reactivation of silenced tumor suppressors offers a mechanism of re-establishing normal cellular signalling and in some cases the induction of apoptosis. One such example is the reactivation of p53 in melanoma, which, though wild-type in 90% of melanomas, is inactivated by aberrant expression of its regulator proteins [36]. A study of compounds that reactivate p53 in melanoma positively identified nutlin-3a as a p53 reactivator and also found potentiation of MEK inhibition upon p53 reactivation [37]. Here, we show that TRIM16 expression induced apoptosis in a range of melanoma cell lines and that the specific expression of TRIM16 with combination treatment was required to induce maximal cytotoxic effect. In addition, TRIM16 expression may potentiate the cytotoxic effect of vemurafenib treatment on BRAF^WT^ melanomas. We found two melanoma cell lines were resistant to combination treatment, both in clonogenicity and cell viability assessment. Interestingly, these two lines showed a markedly higher basal TRIM16 expression compared to the sensitive lines. We hypothesize that these lines may be intrinsically resistant to combination treatment due to already high levels of TRIM16 activation and that the apoptotic action of TRIM16 may be suppressed by other means in these cells.

We have shown previously that high TRIM16 expression correlates with favourable patient prognosis in a cohort of stage III melanoma patients [29]. As combination treatment of vemurafenib and C012 increases TRIM16 protein expression, we investigated the tumor growth rate of engrafted BRAF^WT^/NRAS^Q61R^ melanoma cells, sensitive to the drug combination *in vitro*, in a xenograft model. We observed that the combination of C012 and vemurafenib significantly slowed tumor progression and caused an initial decrease in tumor volume, in agreement with our *in vitro* data. The combination effect is overcome, with the tumor resuming growth and exhibiting the typical adaptability of melanoma to circumvent drug treatment [38]. However, the observation of a significant delay in tumor growth with the combination treatment indicates *in vivo* efficacy and potential for further drug development. Encouragingly, tumor immunohistochemical staining for TRIM16 expression showed a significant increase in the combination treatment group supporting our *in vitro* results which showed that induction of TRIM16 expression was required for the cytopathic effect of the combination. Further studies are required to determine the optimal drug dosing, timing, and pharmacokinetic profile of C012 to gain maximum drug efficacy.

Clinically, vemurafenib is not currently used for the treatment of non-BRAF mutant melanoma due to a lack of efficacy and the paradoxical transactivation of the key driver of melanomagenesis, the MAPK pathway, and the subsequent cell proliferation [39]. However, synergy between vemurafenib and small molecules in NRAS driven melanoma has been described *in vitro*. One study demonstrated a synergistic relationship between metformin and vemurafenib in 7/8 BRAF wild-type/NRAS mutant melanoma cell lines tested [40]. Despite these results, the vast majority of studies investigating novel drug combinations for vemurafenib do not assess BRAF^WT^/NRAS^Q61R^ mutant melanoma cells. Our novel mechanistic insight of the re-activation of TRIM16 in this study provides a basis for further investigation of small molecules that induce TRIM16 for the treatment of metastatic melanoma. It is unknown whether TRIM16 reactivation may potentiate other targeted anti-melanoma therapy and may have a wider application in enhancing drug treatments. It is also worth exploring the possibility of IFNβ1 agonists as anti-melanoma agents as small molecules eliciting the type 1 interferon response have been investigated as candidate anti-viral agents [41] and may have application to melanoma therapy. Furthermore, the recent development of BRAF inhibitors that do not trans-activate MAPK signalling termed ‘paradox-breakers' [42] may synergise with C012 and could offer a combination therapy purposed for BRAF^WT^ melanomas.

Further testing of C012 as a cationic amphiphile is required to assess whether C012 mediates its effect by membrane perturbation or by specific targeting of an as yet unknown protein [43]. Further studies involving optimization of C012 to sub-micromolar levels of activity and structure-activity relationship (SAR) development, correlation of SAR with unbound concentration and efficacy *in vivo* and biomarker (TRIM16) response, would further support a specific action of C012 and validate this compound as a candidate for therapeutic development. Such studies are beyond the scope of the current manuscript but have been initiated and will be reported on in due course.

Overall, our findings offer novel mechanistic insights of potential therapeutic targets in melanoma. Here, we implicate reactivation of TRIM16 as being partially required for tumor targeting by the combination therapy and suggest TRIM16 reactivation as an area for further investigation for melanoma treatment and a potential strategy for targeting BRAF wild-type and mutant melanomas.

## MATERIALS AND METHODS

### High-throughput compound screening

A pilot screen of a library of 10,560 compounds from the Walter and Eliza Hall Institute (Melbourne, Australia), was screened for enhancers of the HDAC inhibitor, suberoylanilide hydroxamic acid (SAHA), at 1.9 μM in a cell-based assay using MDA-MB-231 cells. A total of 352 hit pick compounds (that reduced cell viability < 40%) were selected and screened in the presence or absence of SAHA. 24 compounds maintained < 40% cell viability in the presence of SAHA and > 70% viability in the absence of SAHA (at least 55% difference) giving a hit rate of 0.23%. The 24 hit compounds were assessed for the ability to enhance SAHA in melanoma cells, MM200 and Mel-JD. Three compounds showed single agent anti-melanoma activity. One compound (C012) showed significant synergy when combined with the BRAF inhibitor, vemurafenib, and was selected for further assay.

### Tissue culture, siRNA and plasmid transfection

Melanoma cell lines, CHL-1, IPC-298 and SK-Mel-2 were kindly gifted from Professor Grant MacArthur at the Peter MacCallum Institute, Melbourne. Cell lines, Mel-JD, Mel-RM, Mel-CV, M4405 and MM200 were kindly gifted from Professor Xu Dong Zhang at the University of Newcastle. Melanoma cell lines, A375 and G361 were purchased from ATCC. All lines were cultured in Dulbecco's modified eagle medium (Life Technologies Australia, VIC, Australia) supplemented with 5% foetal calf serum and incubated at 37^°^C/5% CO_2_. TRIM16-1 siRNA 5′AGTAATTCACCATGCAGGTTT-3′, TRIM16-2 siRNA 5′TCTCCCTCCTGCATTTGTGTT-3′ were custom designed and transfected at 20 nM using lipofectamine 2000 as the transfection agent and siControl non-targeting pool (Thermo Scientific, MA, USA) as a control. Smartpool ONTARGERTplus IFNβ1 siRNA was used (Dharmacon, USA). TRIM16 over-expression was achieved by transient transfection of the pcDNA3.1/myc-his tag plasmid containing the full-length TRIM16 cDNA under a CMV promoter using lipofectamine 2000 (Life Technologies Australia, VIC, Australia).

### Cell viability and apoptosis assays

Alamar Blue assays were performed on melanoma cells had been treated with drugs for 72 hours, unless otherwise stated. At the specified time, Alamar Blue was added (1:10) dilution to proliferating cells in the culture media and a baseline colorimetric measurement taken using a Victor 3 multiplate reader. Cells were allowed to proliferate for 4–6 hours at 37°C with 5% CO_2_ incubation, after which time a measurement was taken to record the colour change due to Alamar Blue dye cleavage. The reading was normalized to the baseline reading for each well and colorimetric changes were analysed compared to their respective controls using GraphPad Prism version 6.01.

Apoptosis was measured by the quantification of histone-complexed DNA fragments (mono- and oligonucleosomes) from the cell cytoplasm using a cell death detection ELISA^PLUS^ kit from Roche Applied Science (Mannheim, Germany) as per manufacturer instructions. TdT-mediated dUTP-biotin nick end labeling (TUNEL) (Roche Diagnostics Australia) was used as a secondary confirmation of apoptosis as per manufacturer's instruction and flow cytometry analysis using FACSCalibur flow cytometry (BD BioSciences) as indicated.

### Western blotting and immunohistochemistry

Protein lysate was standardized using the BCA protein quantitation assay kit as per manufacturer's instructions (Thermo Scientific, IL, USA). Western blotting used the following antibodies: polyclonal TRIM16 (Bethyl laboratories, TX, USA), rabbit polyclonal actin antibody (Sigma, St Louis, MO, USA) and anti- GAPDH antibody (Abcam, NSW, Australia). Samples were run on a Bio-Rad criterion (Tris-HCl) 10.5–14% gradient gel (Bio-Rad, NSW, Australia).

Whole tissue sections from excised xenograft tumor were probed with specific TRIM16 antibody at a 1:250 dilution (Bethyl laboratories, TX, USA) and IgG at a 1:500 dilution (Dako, VIC, Australia) was used as a negative control. A rabbit-biotinylated secondary antibody was used at 1:500 dilution (Dako, VIC, Australia). Samples were blindly graded on an arbitrary scale of 0–2 in 0.25 increments, with 2 being the highest staining intensity and 0 as negative staining. Tissues were graded at three different tumor sites to allow for heterogeneity of staining intensity within the tissue. Data were analysed using the Student's *t*-test. Results were considered statistically significant with a *p* value of < 0.05.

### Drug treatment and colony forming assays

Standardized dosing of vemurafenib and C012 was 5 μM and 4 μM, respectively, for BRAF^WT^ cell lines, and 0.5 μM and 4 μM for BRAF^MT^ cell lines. Treatment duration was for 72 hours unless otherwise stated.

For colony forming assays, melanoma cells are seeded at 100 cells/well in 6-well plates. Cells are cultured with control (DMSO only), C012 at 4 μM, vemurafenib at 0.5 μM (BRAF^MT^) or 5 μM (BRAF^WT^) or combination C012/vemurafenib in 2 mL of media. Colonies were allowed to form over 14 days and were then fixed and stained with 2 mL of 6% gluteraldehyde and 0.5% crystal violet solution prepared in Milli-Q water and counted. A colony was determined to be 50 cells or more.

### Pharmacokinetic determination and *in vivo* xenograft study

The pharmacokinetics of C012 was studied in healthy Wistar Rats. Animals were supplied by Laboratory Animal Services, the University of Adelaide, SA, Australia and approval for the study was obtained from the ethics committee of SA Pathology. After overnight fasting, C012 was administrated i.v. (5 mg/kg) *via* the tail vein. Blood samples were collected at 0, 2, 5, 10, 15, 30, 45, 60, 120, 180, 300 and 420 min after dosing. Plasma was separated and stored at −20°C pending analysis. A Shimadzu Nexera HPLC system was used to analyse the sample through a Kinetex C18 1.7 u 50 × 2.1 mm column (Phenomenex, CA, USA) at a mobile phase flow rate of 0.6 mL/min. Mobile phase A (MPA) was 5% acetonitrile and 0.1% formic acid in water and mobile phase B (MPB) was 95% acetonitrile and 0.1% formic acid. The mobile phase was run as a gradient using the following timetable: 0.0–0.5 min 10% MPB; 0.5–3.0 min 10–55% MPB; 3.0–3.1 min 55–95% MPB; 3.1–3.4 min 95% MPB; 3.4–3.8 min 10% MPB. C012 and internal standard were detected by a triple TOF-MS 5600 (AB Sciex, Concord, ON, Canada) in positive ESI mode following the mass transitions: C012 *m/z* 377.2 t7 100.1. Peak areas were obtained from known concentration calibrators of C012 and IS. These were used to construct 2 non-zero 7-point calibration curves from the C012/IS area ratios in the range 5–250 ng/mL and 250–2500 ng/mL respectively. The mean values of concentration at each sampling time point were used to conduct the pharmacokinetic analysis, which was performed with Phoenix^®^ WinNonlin^®^ (Pharsight, Certara^™^, L.P., NJ, USA) using a non-compartmental model.

The maximum tolerated dose (MTD) of C012 in Balb-c nude mice treated at 5, 10, 15, and 20 mg/kg i.v. for a period of 2 weeks, 5 days on/2 days off (total of 10 doses). Signs of toxicity were monitored by body weight and physical symptoms. An MTD of 15 mg/kg was determined and used for the subsequent xenograft study.

Mel-JD cells were engrafted into Balb-(c) nude mice (2 × 10^6^ cells) and tumor volume allowed to reach 200–250 mm^3^. Vemurafenib was administered by oral gavage (p.o.) at 75 mg/kg, C012 was administered by intravenous (i.v.) injection at 15 mg/kg and relevant control vehicles (5% DMSO in PBS, i.v., p.o.). All treatment groups were given for a period of five days on/two days off for a total of 14 days. Tumor volume was measured using callipers every second day and animals were monitored for body weight and physical symptoms. The percentage of tumor growth inhibition was calculated using the following formula %TGI = (1-{Tt/T0 / Ct/C0} / 1-{C0/Ct}) X 100 where Tt = median tumor volume of treated at time t, T0 = median tumor volume of treated at time 0, Ct = median tumor volume of control at time t and C0 = median tumor volume of control at time 0.

## SUPPLEMENTARY MATERIALS FIGURES AND TABLES


