# Supplementary material for: A novel compound which sensitizes BRAF wild-type melanoma cells to vemurafenib in a TRIM16-dependent manner
Source: Oncotarget. 2016 Jul 19;7(32):52166–78. doi: 10.18632/oncotarget.10700 (PMC5239542; doi:10.18632/oncotarget.10700)
Supplement: Supplementary file 1 [file oncotarget-07-52166-s001.pdf]

A novel compound which sensitizes BRAF wild-type melanoma cells to vemurafenib in a TRIM16-dependent manner

Supplementary Materials

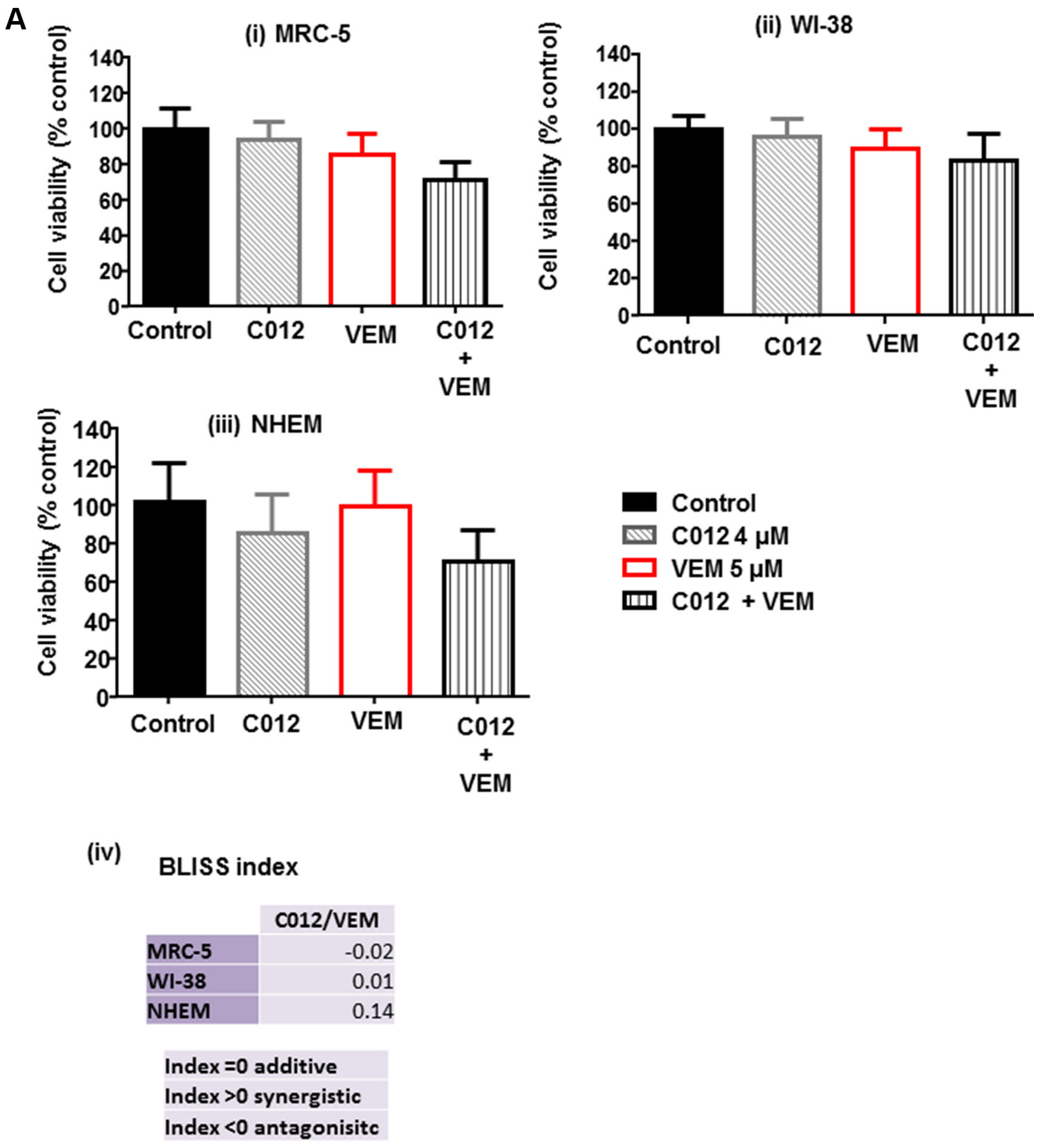

Supplementary Figure S1: Combination of C012 and vemurafenib is minimally toxic to normal fibroblasts and normal melanocytes. (**A**) MRC-5 (i), WI-38 (ii) and NHEM (iii) cells were treated with DMSO control, C012 at 4 μM, vemurafenib (VEM) at 5 μM, or combination (C012 + VEM) for 72 hours and cell viability was assessed by Alamar Blue cell viability assay (iv) Bliss additivity analysis was applied to determine synergy, additivity, or antagonism between C012 and VEM in MRC-5, WI-38 and NHEM cells. Index < 0 is antagonistic, index = 0 is additive, index > 0 is synergistic.

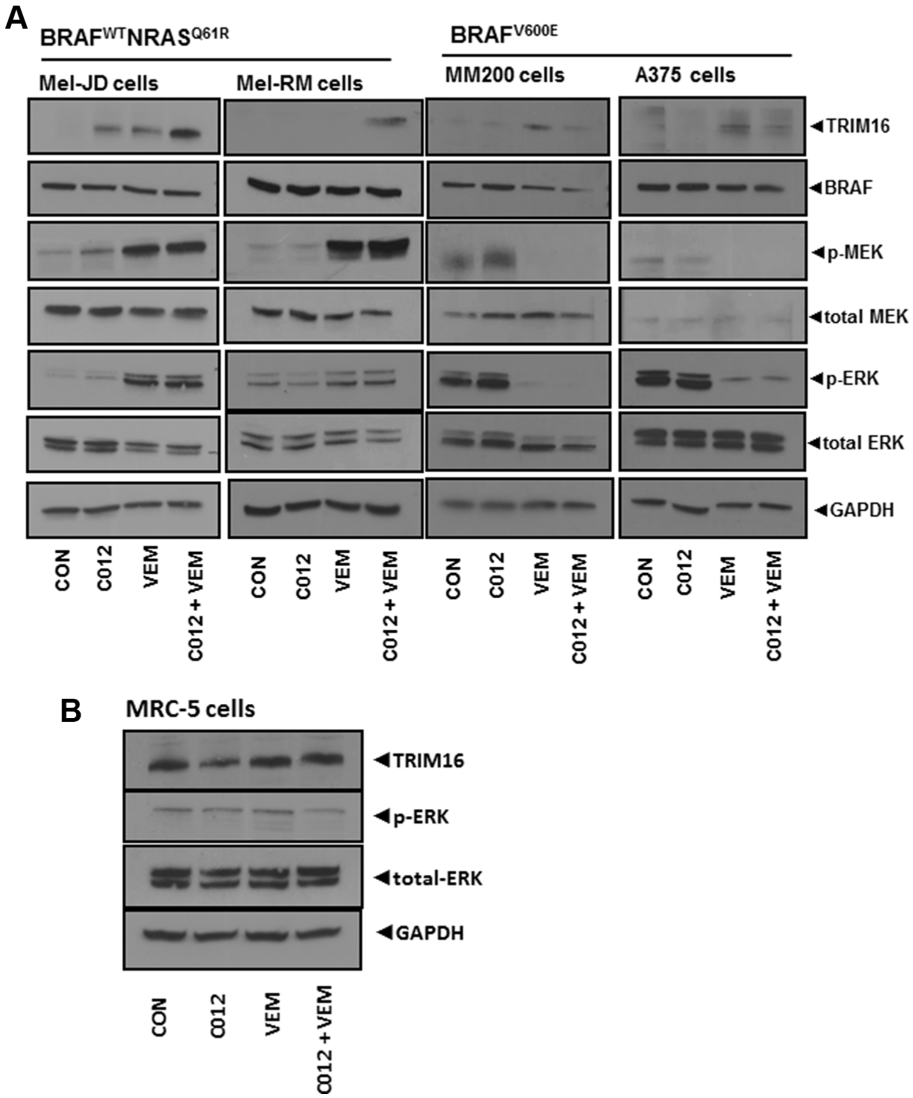

Supplementary Figure S2: TRIM16 is reactivated with combination C012 and vemurafenib in BRAF wild-type, but not mutant cells. (**A**) Mel-JD, Mel-RM, MM200 and A375 cells lines were assessed at 72 hours of treatment of DMSO control, C012 at 4 μM, vemurafenib (VEM) at 5 μM, or combination (C012 +VEM) by western blotting for molecular changes in ERK and MEK phosphorylation for activity assessment of the MAPK pathway. TRIM16 protein expression was also assessed. GAPDH was used as a loading control. (**B**) Immunoblotting was performed for ERK phosphorylation and TRIM16 protein expression with GAPDH is used as a loading control in normal fibroblast line, MRC-5.

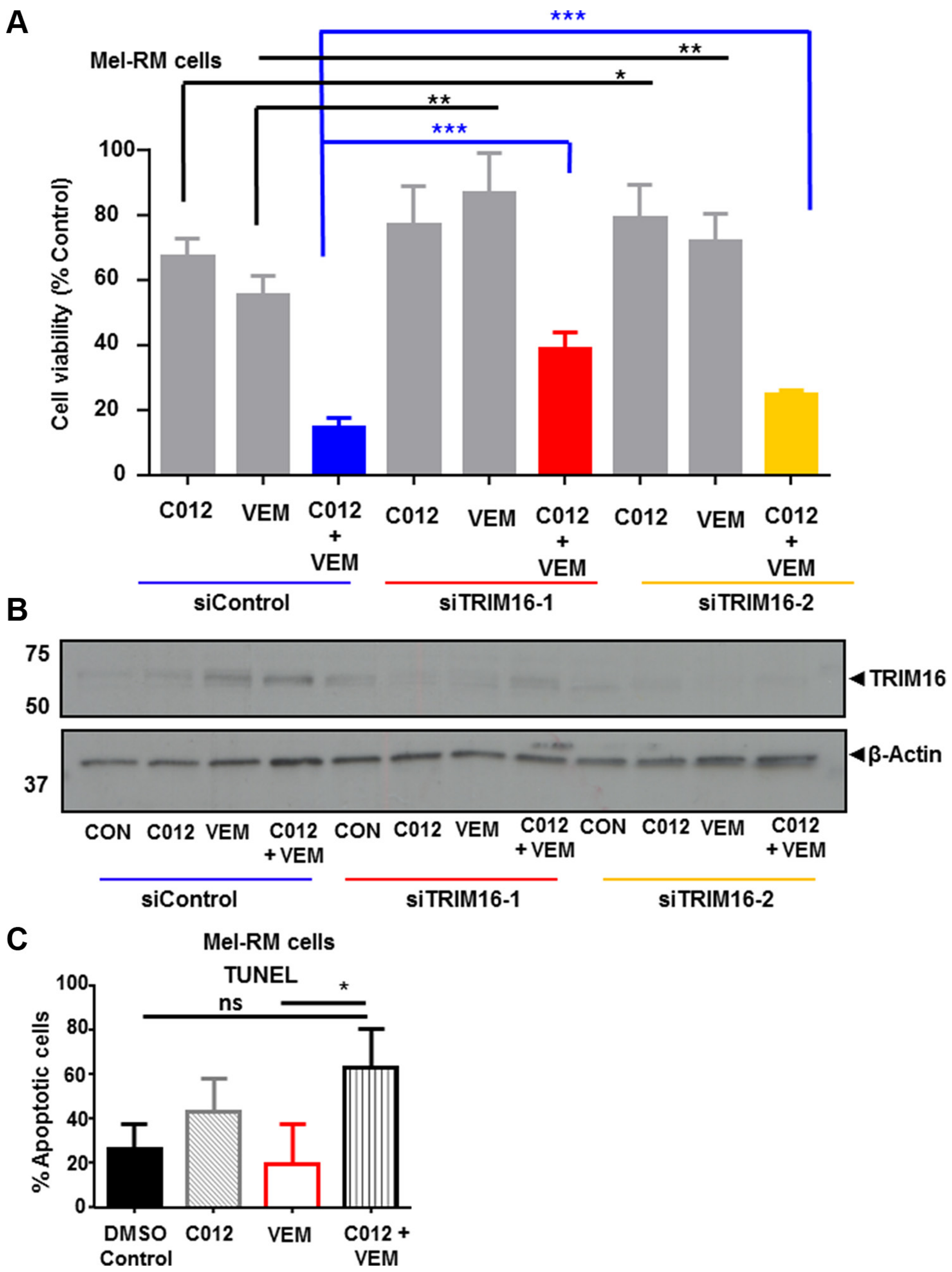

Supplementary Figure S3: TRIM16 is partially required for combination C012 and vemurafenib reduction in cell viability in Mel-RM cells. (**A**) Mel-RM cells were transfected with control siRNA or two specific TRIM16 siRNAs (TRIM16-1 and TRIM16-2) for 24 hours before DMSO control, C012 (4 μM), VEM (5 μM) or combination treatment was added for an additional 48 hours. Cell viability was assessed by Alamar blue assay and expressed as percentage of DMSO control cells. (**B**) Western blotting was used to determine the corresponding protein expression of TRIM16 with GAPDH as a loading control. (**C**) Apoptosis with drug treatment was measured by the TUNEL assay. Statistical analysis was performed by the Student's *t*-test $^{***}P < 0.001$, $^{**}P < 0.01$, $^{*}P < 0.05$.

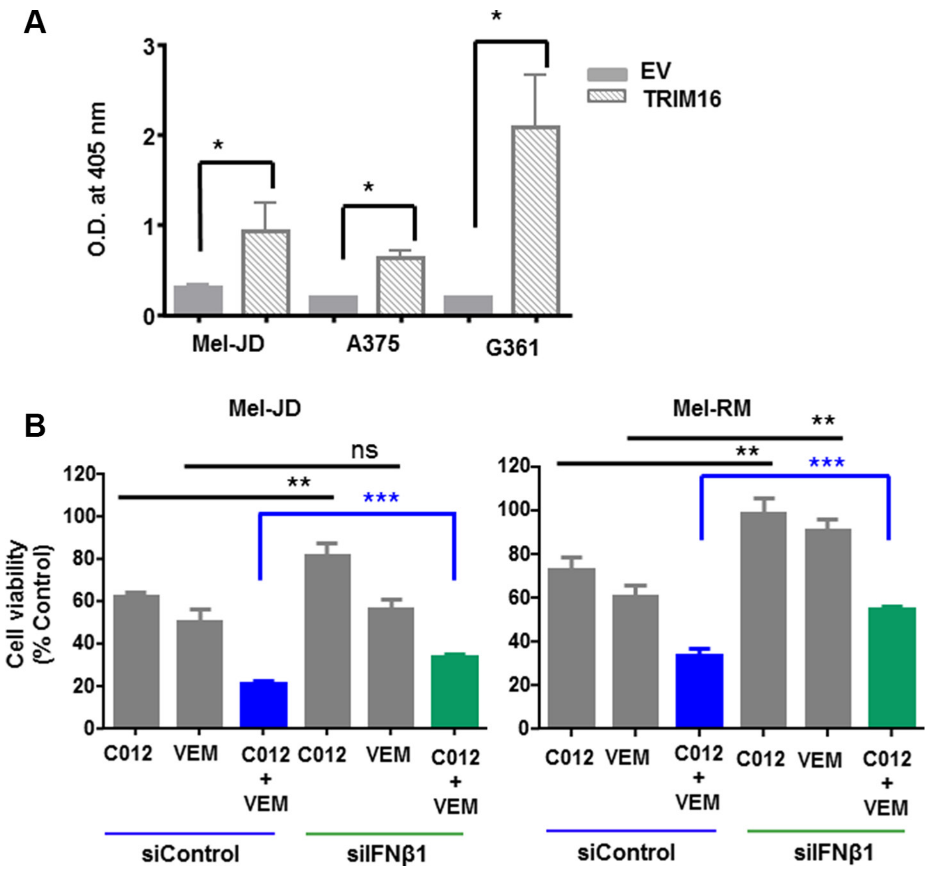

Supplementary Figure S4: IFNβ1 is partially required for combination C012 and vemurafenib reduction in cell viability. (**A**) TRIM16 was overexpressed using a pcDNA3.1 expression vector as an empty vector (EV) or TRIM16 expressing vector (TRIM16) in Mel-JD, A375 and G361 melanoma cells for 48 hours. Apoptosis was measured using an ELISA assay of DNA fragmentation. (**B**) Mel-JD and Mel-RM cells were transfected with control siRNA or IFNβ1 siRNA for 24 hours before DMSO control, C012 (4 μM), VEM (5 μM) or combination treatment for an additional 48 hours. Cell viability was assessed by Alamar blue and expressed as percentage of DMSO control cells. Data was analysed by the Student's *t*-test ***$P < 0.001$, **$P < 0.01$, *$P < 0.05$.

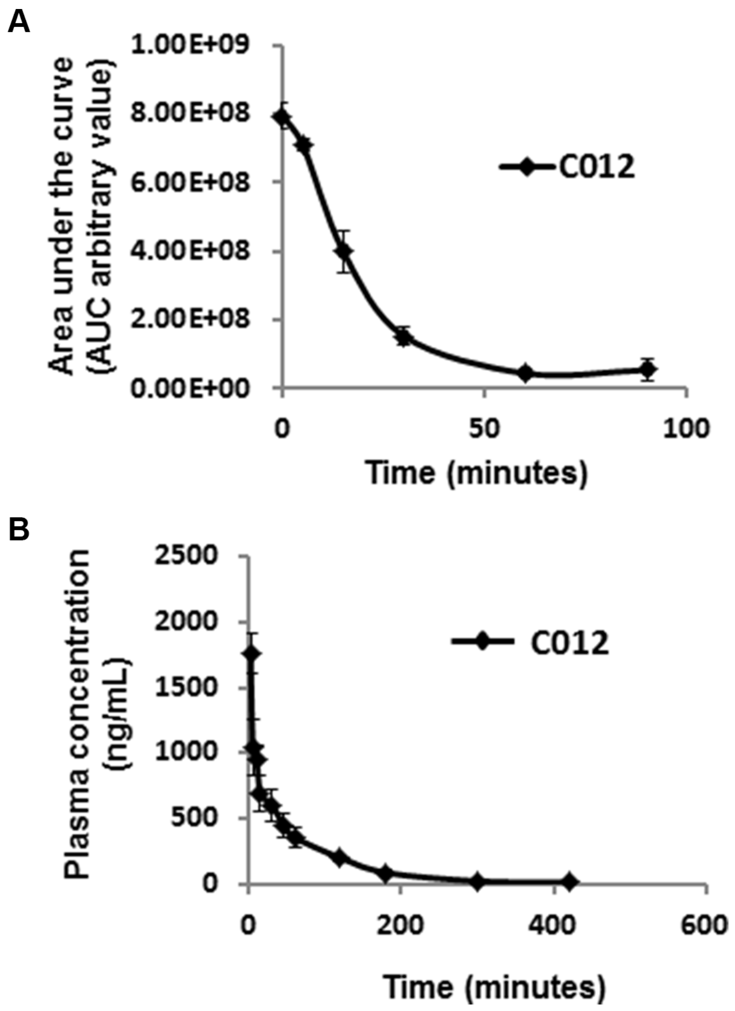

Supplementary Figure S5: Intravenous C012 administration has a favourable half-life. (**A**) Human liver microsomes were incubated with C012 for 5, 15, 30, 60 and 90 minutes and an equal volume of acetonitrile was added to the samples in preparation for mass spectrometry analysis of C012 integrity. Data is expressed as area under the curve (AUC). (**B**) Wistar rats ($N = 3$) were administered a bolus dose of 5 mg/kg C012 intravenously, and blood samples taken at the indicated times for assessment of plasma C012 levels by mass spectrometry. C012 concentration in plasma is expressed in ng/mL.

Supplementary Table S1: The MTD of C012 is determined to be 15 mg/kg. (**A**) The maximum tolerate dose (MTD) of C012 was established by intravenous dose escalation from 5–20 mg/kg of C012 for 14 days (5 days on, 2 days off) and monitoring of animal weight and physical signs. *N* = 3 mice per treatment group. (**B**)Animal weight and physical signs for the last 4 days of treatment are shown. 15 mg/kg is determined as the MTD.

The MTD of C012 is 15 mg/kg

A

Groups	(*n*)	C012 treatment	Completion time
1	3	20 mg/kg i.v. 5 days on, 2 days off	2 weeks
2	3	15 mg/kg i.v. 5 days on, 2 days off	2 weeks
3	3	10 mg/kg i.v. 5 days on, 2 days off	2 weeks
4	3	5 mg/kg i.v. 5 days on, 2 days off	2 weeks

B

C012 dose	Day 10 Appearance and weight	Day 11 Appearance and weight	Day 12 Appearance and weight	Day 13 Appearance and weight
**Groups 1: 20 mg/kg**	moved slowly, 18.7 g	moved slowly, 18.8 g	normal, 19.1 g	normal, 19.0 g
	normal, 20.2 g	normal, 20.3 g	normal, 20.5 g	normal, 20.7 g
	moved slowly, 20.1 g	hunched slightly, 20.3 g	normal, 20.3 g	normal, 20.8 g
**Group 2: 15 mg/kg**	normal, 20.4 g	normal, 20.4 g	normal, 20.6 g	normal, 20.1 g
	normal, 19.8 g	normal, 19.8 g	normal, 20.0 g	normal, 20.2 g
	normal, 21.3 g	normal, 20.1 g	normal, 20.2 g	normal, 20.2 g
**Group 3: 10 mg/kg**	normal, 19.8 g	normal, 20.0 g	normal, 20.3 g	normal, 20.1 g
	normal, 20.6 g	normal, 20.9 g	normal, 20.8 g	normal, 21.0 g
	normal, 21.0 g	normal, 20.9 g	normal, 21.2 g	normal, 20.8 g
**Group 4: 5 mg/kg**	normal, 19.8 g	normal, 20.0 g	normal, 20.4 g	normal, 20.8 g
	normal, 20.2 g	normal, 20.3 g	normal, 20.1 g	normal, 20.3 g
	normal, 21.3 g	normal, 21.5 g	normal, 21.6 g	normal, 22.2 g
